# Accuracy and self-validation of automated bone age determination

**DOI:** 10.1038/s41598-022-10292-y

**Published:** 2022-04-16

**Authors:** D. D. Martin, A. D. Calder, M. B. Ranke, G. Binder, H. H. Thodberg

**Affiliations:** 1 University of Witten/Herdecke, Witten, Germany; 2 Pediatric Endocrinology, University Children’s Hospital, Tübingen, Germany; 3 Great Ormond Street Hospital for Children NHS Foundation Trust, London, UK; 4 Visiana, Fremtidsvej 1, 2970 Hørsholm, Denmark

**Keywords:** Computational biology and bioinformatics, Endocrinology, Mathematics and computing

## Abstract

The BoneXpert method for automated determination of bone age from hand X-rays was introduced in 2009 and is currently running in over 200 hospitals. The aim of this work is to present version 3 of the method and validate its accuracy and self-validation mechanism that automatically rejects an image if it is at risk of being analysed incorrectly. The training set included 14,036 images from the 2017 Radiological Society of North America (RSNA) Bone Age Challenge, 1642 images of normal Dutch and Californian children, and 8250 images from Tübingen from patients with Short Stature, Congenital Adrenal Hyperplasia and Precocious Puberty. The study resulted in a cross-validated root mean square (RMS) error in the Tübingen images of 0.62 y, compared to 0.72 y in the previous version. The RMS error on the RSNA test set of 200 images was 0.45 y relative to the average of six manual ratings. The self-validation mechanism rejected 0.4% of the RSNA images. 121 outliers among the self-validated images of the Tübingen study were rerated, resulting in 6 cases where BoneXpert deviated more than 1.5 years from the average of the three re-ratings, compared to 72 such cases for the original manual ratings. The accuracy of BoneXpert is clearly better than the accuracy of a single manual rating. The self-validation mechanism rejected very few images, typically with abnormal anatomy, and among the accepted images, there were 12 times fewer severe bone age errors than in manual ratings, suggesting that BoneXpert could be safer than manual rating.

## Introduction

The bone age—or skeletal maturity—of a child expresses the age of the child according to its own biological clock. It is determined from a radiograph of the child’s hand by comparing it to an atlas of images typical of children at different ages^1^. Bone age is an important piece of information in the diagnosis and treatment of a range of disorders addressed in paediatric endocrinology^2,3^. Automated determination of bone age using a computer has been used routinely in clinical practice since 2009, when the BoneXpert method was introduced^4^, and today more than 200 radiology departments are using the method integrated in their digitalised workflow.

This paper presents a major upgrade of the method to version 3 that improves the algorithm, extends the bone age range down to neonates, and adds a separate analysis of carpal bone age.

The aim of the paper is to introduce the new version and evaluate its *accuracy* and the *self-validation*. The primary audience is radiologists and pediatric endocrinologists who want to critically assess the method. Therefore, the paper focusses on reporting validation studies. The paper will also be of interest to medical device specialists and AI developers, who will appreciate the more technical information on the algorithm found in the supplementary material.

### Accuracy

The *accuracy* of a measurement method is defined as its agreement with a suitably defined *gold standard*, defining the true value, and hence accuracy is sometimes called *trueness*. In this work, we define the *true* bone age of a hand X-ray as the average of the ratings by very many experts using the Greulich-Pyle (GP) atlas method^1^. This definition is operational: For any given X-ray, one can determine bone age as close to the truth as desired, by asking sufficiently many operators to rate the image.

A challenge with this definition is that the standard deviation (SD) of manual ratings of the same image is quite large—for instance it was found to be 0.58 years (y) in a study with 12 raters^5^. The large interrater variability is a main motivation for adopting an automated method.

When comparing an automated method with a single routine manual rating on images taken and rated as part of clinical practice, the observed Root Mean Square (RMS) error comprises contributions from both the inaccuracy of the automated method and the human rater variability. To disentangle these, the images can be rated independently by *multiple* raters. This was accomplished elegantly in the Bone Age Machine Learning Challenge organised by the Radiological Society of North America (RSNA) in 2017^6^. RSNA published 14,036 hand images with a single bone age rating and invited teams to develop an algorithm to predict the bone age from an image. To benchmark the competing methods, RSNA created a test set of 200 images with *six* independent manual ratings. The SD among the six ratings was 0.68 y, and the *reference rating* was defined as the average of the six. The SD between the *reference* and the *true* rating can be estimated as 0.68 y/sqrt(6) = 0.31 y. RSNA preferred to use the Mean Absolute Deviation (MAD) rather than the RMS error; following RSNA, we therefore also use the MAD. The reference had a MAD of 2.8 months relative to the true rating. A total of 48 Challenge participants submitted predictions on the 200 test cases, and they were evaluated by their MAD from the reference rating. The best five methods had MAD in the range 4.3–4.5 months. The performance of these five were not significantly different, so they were declared as winners, and the five methods were presented in a joint publication^6^. The RSNA test set has become the de facto benchmark for bone age methods, and it has helped boost the accuracy of new methods^7^.

In this paper, we evaluated the accuracy of the new version 3 in two ways: Firstly, we assessed the RMS error of version 3 relative to a single rater in a very large study and compared with the performance of the previous version. Secondly, we evaluated the accuracy on the RSNA test set against the average of the six ratings.

### Self-validation

Automated bone age is an example of artificial intelligence (AI). We distinguish between AI systems that *assist* the human, **AI-Assist**, and AI systems capable of completely *replacing* the expert, **AI-Replace**. BoneXpert was designed to be able to work as AI-Replace. However, in a recent survey among radiologists using BoneXpert^8^, it was found that 83% of the users are still looking at the bone age X-ray, and that the main reason for this is to check for signs of underlying disease, such as skeletal dysplasia; the BoneXpert bone age value is changed only rarely: 40% of the users never change it, and 43% change it in less than 5% of the cases.

When the rating of bone age is taken over by an automated method, there is a risk that the image is outside the range of validity of the method. This risk is obviously present if the method is used as AI-Replace, but even when used with supervision, the risk remains, because one cannot expect that the user knows the limitations of the software. This risk was addressed in the design of BoneXpert, by equipping it with a *self-validation mechanism* that automatically omits the bone age assessment if the image is outside its range of validity, as described in detail in the Methods section.

We assessed the self-validation mechanism in two ways. Firstly, we report how often an image was rejected in the large RSNA data set, and we illustrate the rejection mechanism by four examples of accepted hand X-rays on the border of being rejected, i.e. they had many rejected bones.

Secondly, we searched a large set of *accepted* images for cases with potentially incorrect bone age assessment, by selecting the subset of *disputed* images, defined as cases where the BoneXpert rating deviated by at least 1.8 y from the manual rating. These were rerated blindly by three raters and the average defined as the *reference rating—*a proxy for the true rating. This allowed us to examine whether the disputed cases were due to an inaccuracy of BoneXpert, or due to a manual rating error. This treats manual and automated ratings on an equal footing and allowed us to answer the question: who is most likely to make a gross error: the human or the machine, and what are these error rates?

## Materials and methods

### Material

The following study data were used for the training of the bone age model. They had one manual rating per image, unless indicated otherwise:

- *The Paris longitudinal study*, including 2600 images from 410 normal children born in 1953–57, followed with X-rays at ages 1, 3, 6, 9, 12, 18 months and then at ages 2, 3, 4 etc. We included images up to the age of 12. There were no manual ratings^9^.
- *The RSNA training and validation sets*, including 14,036 images from clinical practice at Lucile Children’s hospital in Stanford and Colorado Children’s hospital^6^.
- *The Tübingen Short stature study*, including 6743 images from 1197 children with a range of Short Stature diagnoses: Growth Hormone Deficiency (40%), Turner syndrome (20%), Small for Gestational Age (8%), Silver-Russell Syndrome (3%), Noonan Syndrome (2%), Idiopathic Short Stature (2%) and others (25%). One sixth of the study was previously used to validate the previous version of BoneXpert^10^.
- *The Tübingen Congenital Adrenal Hyperplasia study*, including 775 images from 100 children, also used to validate the previous version^11^.
- *The Tübingen Precocious Puberty study*, including 732 images from 116 children, also used to validate the previous version^12^.
- *The Erasmus study,* including 542 images of normal children from Rotterdam 1997^13^, also used to validate the previous version^14^.
- *The Los Angeles study*, Including 1103 images of normal children of four ethnicities from 1993–2006^15^, also used to validate the previous version^16^. The manual ratings were formed as the average over two raters.

The part of the algorithm analysing radius and ulna at the end of puberty had already been developed using the following data:

- *End of Puberty study,* including 3500 images of normal subjects followed longitudinally and having no bone age rating^17^.

The total number of images used for developing version 3 was 30,031, coming from seven different sites and rated by raters from both Europe and USA. The Tübingen images were recorded in 1972–2004 with a range of different types of equipment. In this way, the trained method is more likely to be valid across equipment, and it will tend to rate as the average over many raters.

Finally, the following study was used to test the developed algorithm:

- *The RSNA test set* of 200 images from clinical practice with a “reference rating” formed as the average over six independent ratings.

We will present results for a combined study, comprising the three Tübingen studies. It was used for a comparison of the RMS error in version 3 and the previous version, and for the study of disputed cases described in the introduction.

We will also split the Los Angeles data into its four ethnicities to assess the accuracy in each of these.

### Informed consent and ethics approval

The Paris study was initiated as a prospective auxological study in 1953 in accordance with the ethical requirements at the time^18^. Informed consent was given by the parents at the birth of the children, who were then followed regularly with X-rays images and auxological measures for up to 18 y. Four similar studies were initiated at the same time, organised as a joint research effort by the International Children’s Centre in Paris.

The RSNA data were published by the RSNA. The Institutional Review Boards at Stanford University and the University of Colorado approved the curation and use of pediatric hand radiographs for the purposes of developing machine learning methods. Patient informed consent was waived after approval by the Institutional Review Boards^6^.

The three Tübingen studies were obtained during routine controls, not study-driven, and the use for developing bone age methods was approved by the Tübingen University Hospital Ethics Committee, which also stated that informed consent by each participant/guardian was not needed.

For the initial Erasmus study^13^, the Institutional Review Board of the Erasmus Medical Center in Rotterdam gave approval to obtain radiographs of the left hand in all children, and the subsequent use of these data for development of bone age methods was permitted by the Board, which also waived the need for informed consent.

Finally, the Los Angeles study is a subset of the publicly available Digital Hand Atlas developed by the University of Southern California Image Processing and Informatics Laboratory in Los Angeles^15^. This retrospective study was approved by the Institutional Review Boards of the Department of Radiology in Los Angeles County Hospital and the University of Southern California Medical Center in Los Angeles, and written informed consent was obtained from all subjects or their guardians.

All methods were carried out in accordance with relevant guidelines and regulations.

### Method: the software architecture

**Figure 1:**
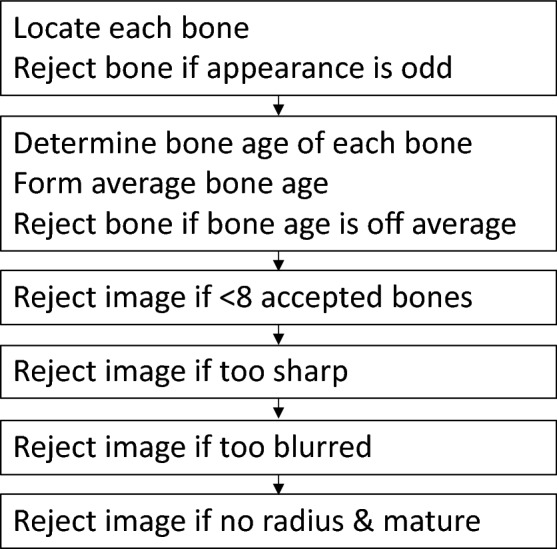
Flowchart of the software architecture. The architecture is dominated by the six steps of the selfvalidation. The first two steps validate individual bone contributions, while the last four validate the entire image.

**Figure 2:**
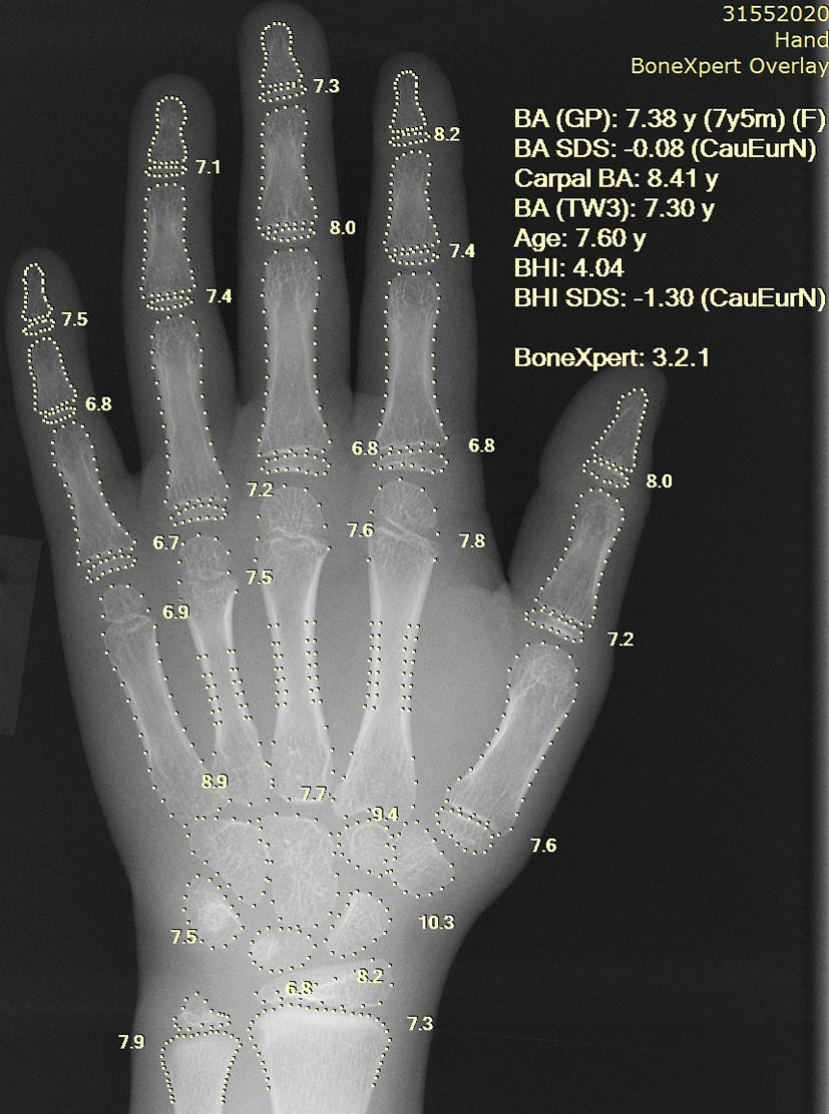
Posterior-anterior left-hand radiograph with bone age assessment by BoneXpert. This is the result file automatically placed in the same study in the digital archive. BA = bone age, averaged over the 21 tubular bones; BHI = Bone Health Index; SDS = standard deviation score; GP = Greulich-Pyle; TW3 = Tanner-Whitehouse Version 3.

The software architecture is shown in Fig. 1. BoneXpert analyses the hand image by performing an examination of each bone with the result shown in Fig. 2. The architecture has six steps, and each step contributes a piece of the selfvalidation. The first two steps analyse the bones individually and can result in the rejection of some bones, while the last four steps perform an overall analysis which can result in the rejection of the entire bone age analysis.

The supplementary material contains details on how the borders of the bones are determined and how the bone age value for each bone is modelled. This is done using conventional image analysis based on machine learning. We use separate bone age models for males and females, except for bone age below 2 y, and for the carpal bone age.

The self-validation is the most novel aspect of BoneXpert, so it warrants a more detailed description. The individual bone analyses are validated in two steps:

A. The bone is rejected if its appearance (shape and bone density distribution) is at odds with what is expected from the bone-finding model, which was training on data containing bones without severe abnormalities.
B. If the bone age of the bone deviates by more than some threshold from the average bone age determined from all the tubular bones, the bone is rejected. The threshold is set at 2.4 y above 7 y, while at lower bone ages it decreases linearly to 1.2 y at birth.

The next phase of the self-validation addresses the overall analysis and can result in four types of rejections:

1. If less than 8 bones survive the above self-validation, the image is rejected as “hand not found”. The limit value 8 is a compromise between safety and efficiency—any value from 5 to 11 would also have merit.
2. The algorithm computes a measure of the sharpness of the image by analysing the steepness of the edges of the three middle metacarpals. This sharpness measure is dimensionless and invariant to linear changes of brightness and contrast. For images recorded on film, the average sharpness lies in the interval 5–6.5. If the sharpness exceeds 13, the image is rejected as “too sharp” – this is due to excessive image postprocessing (edge enhancement).
3. If the sharpness is below 4, the image is rejected as “too blurred”. This happens for instance if the image was recorded on a low-resolution imager, or if the hand moved during the exposure.
4. If the average bone age in the short bones is above 17 y for boys (15 y for girls), the radius is needed for a reliable bone age assessment, because the radius is the only bone showing changes in maturity all the way up to 19 y of bone age (in the case of males)^17^. Therefore, if the radius was rejected in such almost mature images, the entire analysis is rejected as “no radius / mature”.

The last three rejections, type 2, 3 and 4, can be bypassed if the user is willing to suffer a somewhat poorer accuracy, rather than losing the analysis completely – this is useful e.g. in retrospective research studies, and in benchmark studies, where all images must be analysed. However, in clinical practice, the last three conditions lead to rejection of the automated analysis, to ensure the safety of the medical device. The number of rejections of type 2–4 can be reduced to almost zero by adopting a suitable image post-processing protocol and by ensuring that at least 3 cm of radius is visible.

## Results

### Accuracy results

#### Accuracy on training set

We first present, in Table 1, results on the accuracy of BoneXpert on the six studies used for training the model, and we make the following observations:

1. The RMS errors were smallest in the Los Angeles study, which could be because the manual ratings were the average over two ratings.
2. The accuracy is similar across the three disorders studied in Tübingen.
3. The three Tübingen studies showed smaller errors than the RSNA study, which suggest that the manual ratings in Tübingen were more accurate.
4. The Los Angeles data set contained 25% of each of the four ethnicities: Caucasian, African-American, Hispanic and Asian – and there was a higher RMS error in African-American females.

**Table 1:** Accuracy of the BoneXpert bone age method on six studies.

Study	Number of images	RMS error (y)		
		Boys	Girls	Both sexes
Radiological Society of North America	14,036	0.67	0.69	0.68
Tübingen Short Stature	6743	0.62	0.61	0.61
Tübingen Congenital Adrenal Hyperplasia	775	0.57	0.54	0.56
Tübingen Precocious Puberty	732	0.56	0.59	0.58
Erasmus	542	0.64	0.69	0.66
Los Angeles, all ethnicities	1103	0.53	0.57	0.55
Los Angeles, Caucasian	276	0.51	0.48	0.50
Los Angeles, African-American	275	0.54	**0.75**	0.65
Los Angeles, Hispanic	279	0.55	0.52	0.54
Los Angeles, Asian	273	0.53	0.49	0.51

The table presents RMS errors obtained when training on these data; cross-validated RMS errors are 0.01 y higher (see supplementary material). The accuracy in the Los Angeles study is also subdivided into the four ethnicities, and the accuracy is significantly worse in African American girls: It is 0.75 y with a 95% confidence interval of [0.65; 0.85] y.

**Figure 3:**
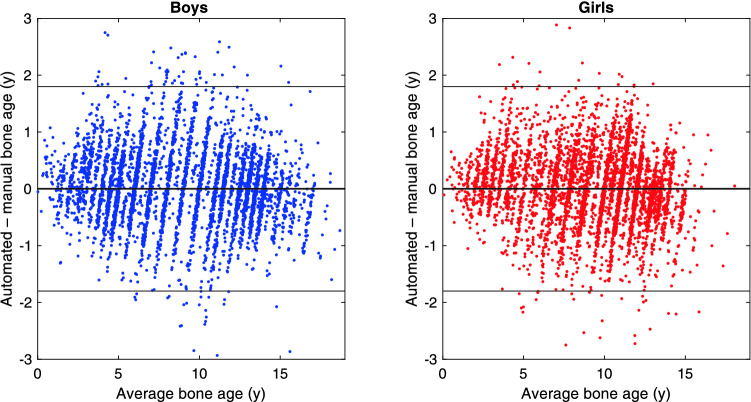
The agreement between automated and manual rating in the three Tübingen studies of children with Short Stature, Congenital Adrenal Hyperplasia or Precocious Puberty. The manual ratings are the original manual ratings without any corrections. The lines at ± 1.8 years define the *disputed* cases to be rerated 3 times. On the horizontal axis is the average of manual and automated bone age rating.

We next present results for the three Tübingen studies combined, which gives a data set with 8250 images. Figure 3 shows the Bland–Altman plot of the agreement of BoneXpert and manual rating on the Tübingen studies. We see that these studies cover the bone age range 0–17 y for males and 0–15 y for females and that outliers are most frequent in the range 7–12 y.

The RMS error averaged over the two sexes is 0.61 y in the Tübingen data. As explained in the supplementary material, we also did a cross-validation to estimate the error on the cases when they are *not* part of the training data, and the result on the Tübingen data is a slightly larger RMS error of 0.62 y. This can be directly compared to the RMS error of the previous BoneXpert version on the same data, which was 0.72 y^10–12^. The new version is significantly better with *p* < 10^–6^.

#### Accuracy on test set

**Figure 4:**
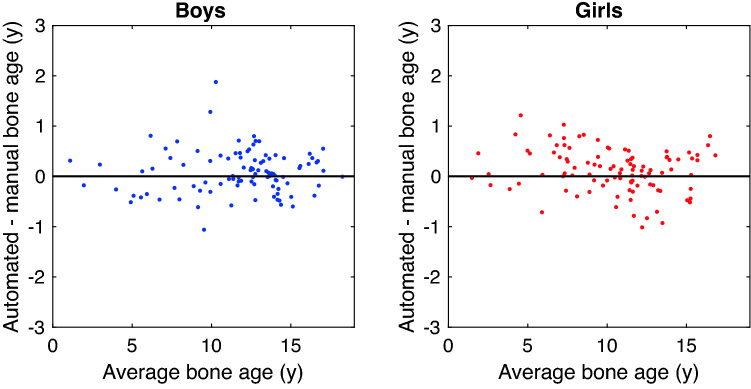
Bland Altmann plot for the agreement between the automated rating and the manual rating for the 200 cases in the RSNA test set.

We now turn to the strongest validation set: the independent RSNA test set with six manual ratings. The agreement between BoneXpert and the reference (the average manual rating) is shown in Fig. 4, with RMS error 0.45 y and MAD 4.1 months. This is a non-significant improvement over the five ‘winners’ of the RSNA Pediatric Bone Age Machine Learning Challenge, which were in the range MAD 4.3–4.5 months. The small RMS error is understandable, as the manual rating here is formed as the average over six ratings, eliminating a large part of the human rater variability. As mentioned in the introduction, the reference has a RMS error of 0.31 y with respect to the true rating, so we can estimate the RMS error of BoneXpert with respect to the true rating as sqrt(0.45^2^–0.31^2^) y = 0.33 y. This “true accuracy” is the ultimate answer to the question of how accurate the BoneXpert method is, as it is independent of any human rater variability.

From the true accuracy we can estimate the rater variability for the Tübingen raters as sqrt(0.62^2^–0.33^2^) y = 0.52 y, which is considerably smaller than the 0.68 y observed for raters in the RSNA study.

#### Accuracy at low bone age (< 2y)

BoneXpert version 3 has extended the bone age range down to neonates, whereas the previous version only reached down to males of bone age 2.0–2.5 y and females down to 1.5–2 y. It is therefore particularly relevant to validate the accuracy of the new version at the lowest bones ages and here we present a validation in the bone age range 0–2 y.

**Figure 5:**
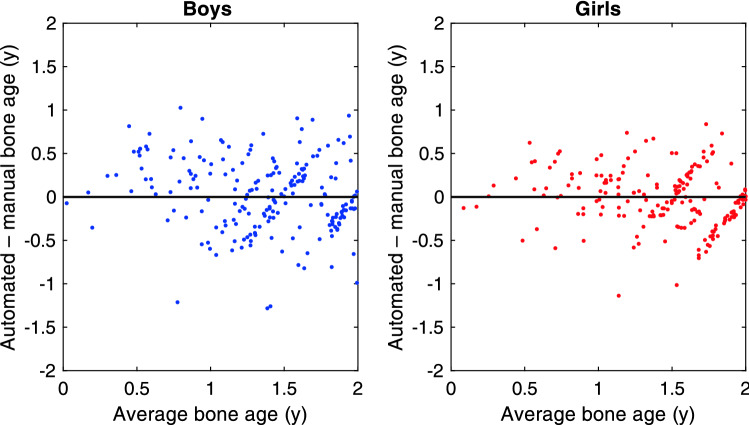
Bland Altman plot of the agreement between automated and manual bone age rating below 2 y.

As detailed in the supplementary material, the bone age models below 2 y were trained on the Paris study, so we can use the RSNA, Tübingen and Los Angeles studies for an *independent* validation of the accuracy of the bone age determination below 2 y. Figure 5 shows the Bland–Altman plot for the agreement between BoneXpert and manual bone age in this range.

The RMS error is 0.41 y for boys and 0.33 y for girls below 2 y. The RMS error for the whole bone age range averaged over genders is 0.61 y, so the observed RMS errors below 2 y are much smaller. The better accuracy can be understood as a result of maturity indicators changing more rapidly at these low ages. The supplementary material presents more details of this analysis.

It is concluded that the accuracy of BoneXpert below 2 y is considerably better than at higher bone ages, although the validation below 0.5 y is rather scarce.

### Self-validation results

The evaluation of the self-validation mechanism was done in two parts. First, we looked at the frequency and the nature of the rejections, and second, we searched for errors of the method among cases that were accepted by the self-validation.

#### Rejected images and bones

In the RSNA study, the self-validation rejected 59 cases, corresponding to 0.4%, due to too few accepted bones, i.e. less than 8 bones (rejection type 1). These cases exhibit clearly abnormal bone structure, wrong pose or poor image quality. In addition, there were 80 cases with rejection type 2, “too sharp”; such cases can be eliminated by adopting a milder post-processing protocol, a common adaptation made when installing BoneXpert in clinical practice. There were no rejections due to blur (type 3) and four rejections due to “no radius/mature” (type 4).

**Figure 6:**
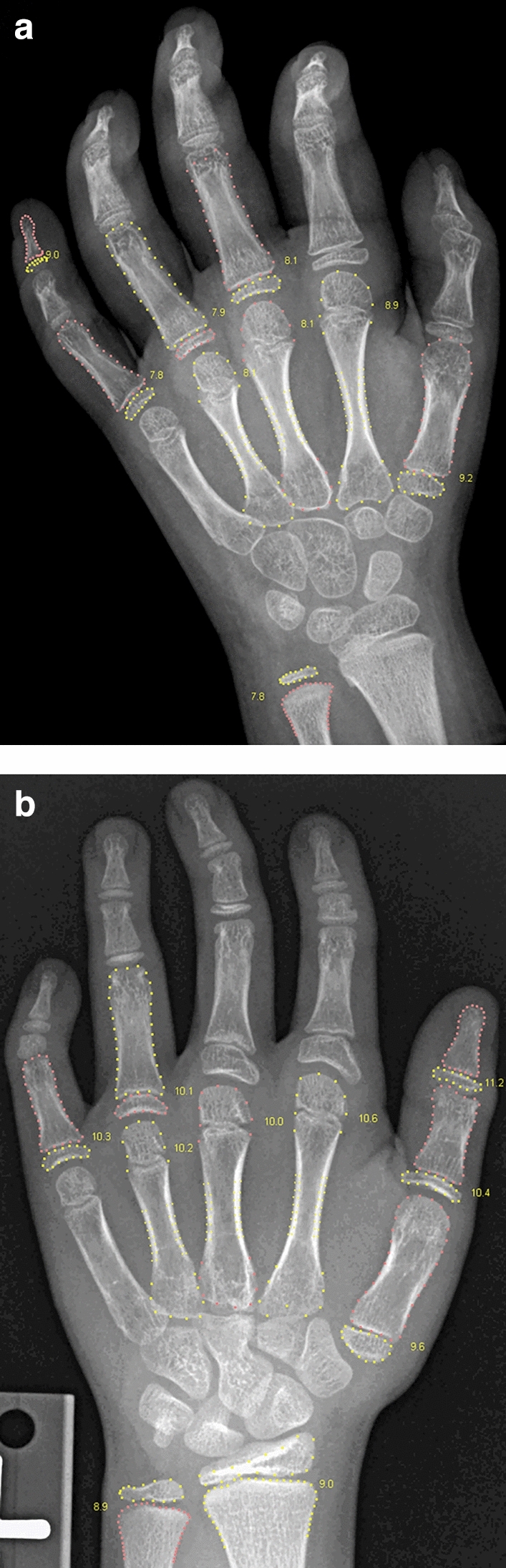
Females 2707 and 3781 from the RSNA study. The automated method accepted only 9 and 10 of the 21 tubular bones, which was just above the 8 bones needed for the entire analysis to succeed. In the first image, the overlap between radius and lunate is likely to have caused radius to be rejected; and the pose of the middle and distal phalanges in finger 2–4 is incorrect due to flexion. In the second image, there is brachydactyly in the second and fifth middle phalanges, and the second and third proximal phalanges have thick epiphyses, suggesting the monogenic disorder Brachydactyly Type C. For clarity, the bone contours are drawn in alternating colours. The numbers indicate the GP bone ages.

**Figure 7:**
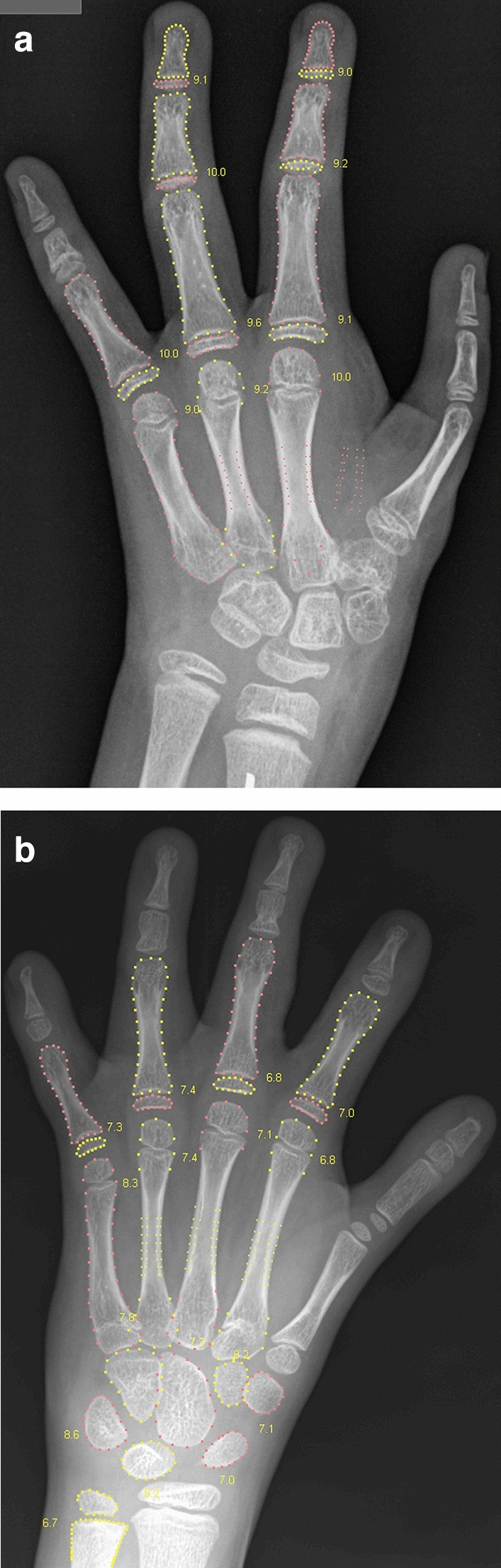
Two females, 4210 and 13,824, from the RSNA study with 10 and 9 accepted tubular bones. Ray 2 is missing in the first image, which causes the Bone Health Index analysis to fail (indicated by the code 3). The radial epiphysis is abnormally thickened and there is pronounced positive ulnar variance. The first digit is likely to be a pollicised index finger following surgery for thumb aplasia or severe hypoplasia; a likely case of radial longitudinal reduction defect. The second image shows brachydactyly in the middle phalanges. Finger 1 has two epiphyses in the metacarpal, and either a thick epiphysis in the distal phalanx or an extra short phalanx; the thumb is somewhat ‘finger-like’. The second and fifth digits similarly appear biphalangeal with large epiphyses of the distal phalanges. Digits 3 and 4 show middle phalangeal hypoplasia. Appearances suggest an underlying genetic disorder of hand-development requiring further evaluation.

Figures 6 and 7 illustrate the self-validation of the individual bones. In the RSNA data, there were 29 cases with 8–10 accepted bones, i.e. cases which were close to being rejected, and four of them are shown in these figures. It is seen that the rejected bones do indeed exhibit abnormal structure or wrong pose. This exemplifies what the method accepts and what it rejects. It is rare to see so few accepted bones: 96.3% of the RSNA images had 19, 20 or 21 accepted bones.

#### Disputed cases

The second way that we evaluated the self-validation, was to look for errors among the *accepted* images in the three Tübingen studies. If the self-validation fails to reject a case where it has assigned wrong bone age, it will almost always present itself as a case with a large deviation from the manual rating, because it is unlikely that the manual rating makes the same error. We selected the *disputed case*s, defined as those where the BoneXpert and manual ratings deviated by more than 1.8 y, as illustrated by the lines in Fig. 3. This resulted in 121 disputed cases. We set the threshold sufficiently low to get at least 100 disputed cases for this investigation. These cases were rerated by three raters (the authors from Tübingen: DDM, MBR, GB), which were blinded from the original and BoneXpert ratings, and from each other, and from the age. The average of the three ratings was defined as the *reference*, which is considered our best estimate of the true rating. We found that for 103 of the cases, or 85%, BoneXpert was closer to the reference than was the original manual rating. As another way of looking at this, we found that 72 of the original manual ratings deviated by more than 1.5 y from the reference, while only 6 of the BoneXpert ratings did.

This analysis of disputed cases has no preference for manual or BoneXpert rating. The analysis is equally sensitive at finding errors in the manual and the automated methods, and we observed that the rate of grave errors is 12 times lower for BoneXpert than for the manual rating, suggesting that BoneXpert is safer than manual bone age rating, even in a hospital like Tübingen with its relatively high reliability of manual bone age rating.

We can understand that human raters had a larger frequency of errors above 1.5 y than BoneXpert from the fact that the accuracy of the Tübingen raters is 0.52 y, while the true accuracy of BoneXpert is 0.33 y. A distribution with wider SD is also expected to have more cases with errors above 1.5 y. The 121 disputed cases constituted 1.5% of the Tübingen study, and most of them were due to manual rating errors. In the RSNA study, 2.4% of the cases (that is 330) deviated by more than 1.8 y, and we do indeed expect a larger error frequency here, because the manual raters in the RSNA data were found to have a larger rating error than in Tübingen, namely 0.68 y.

## Discussion

### Improved accuracy

The accuracy of BoneXpert against a single manual rating was found to 0.62 y compared to 0.72 y in the previous version. We attribute this improvement to three factors: (1) Adding more bones, (2) improving the accuracy and robustness of the localisation of the bone borders, and (3) the much larger training set for the bone age model, which allowed the new model to fit closer to how humans rate using the GP atlas.

The analysis of disputed cases showed that BoneXpert version 3 was more correct than manual rating in 85% of the disputed cases. This is an improvement compared to the previous version, which was more correct in 2/3 of the cases on the same Tubingen data^10–12^—this improvement is attributed to the same reasons as listed above for the improved accuracy.

The accuracy of BoneXpert on the RSNA test set was found to MAD 4.1 months. Many deep learning-based bone age methods have been presented starting with the RSNA Challenge in 2017^6^. The most thoroughly validated method is the one from Stanford^19^, which achieved a MAD of 4.9 months on the RSNA test set.

To appreciate the clinical relevance of a more accurate bone age assessment, we note that the SD of bone age of healthy subjects at a fixed age is of the order of 1.0 y (above the age of 7 y), i.e. the natural *variance* is 1.0 y^2^. The typical manual rater accuracy is 0.58 y, which corresponds to a variance of 0.34 y^2^. This is 34% of the natural variance, which is a large fraction, that limits the usefulness of manual rating. BoneXpert’s true accuracy 0.33 y corresponds to a variance of 0.11 y^2^, which is only 11% of the natural variance, so the reduction of accuracy error is clinically relevant.

### Importance of self-validation

This paper has covered the accuracy of automated bone age assessment in a broad sense: In addition to assessing the accuracy on the 200 images from RSNA, we have addressed the risk of making large errors. Management of this risk was an integral part of the design of BoneXpert, which was constructed to *positively* know when it is inside its own limitation. This enables it to be used as an autonomous method, but even if used with supervision, we argued that such a self-validation mechanism is needed for safety, and we have shown that this mechanism effectively keeps the rate of large errors very low, without causing more than 0.4% rejections.

Risk management throughout the lifecycle of medical devices has become a requirement with the new European Medical Device Regulation^20^, so an AI-based medical device, which is accurate in some studies, but has unmitigated risks of making large errors, is unsuited for use in clinical practice. Such errors are typically relatively rare under ideal conditions, and they might not affect the overall error measure very much, in particular when using an error measure like the MAD. Thus for the RSNA test set, if we change the error of a case from 4 months to 2 y, the RMS error increases by 4.8%, while the MAD changes by only 2.4%; hence our preference for RMS over MAD as a measure of accuracy.

AI systems are new tools in radiology, and understandably doctors are unsure about their limitations. As an example, a recent paper^21^ examined a deep learning bone age assessment method on a wide range of images, including shoulder and thorax images, and the method processed these images with no warning and gave completely wrong bone ages – the authors were critical about the system’s total lack of understanding of its own limitation. This study demonstrates that one cannot expect that radiologists can judge whether images are within the range of the AI method’s validity – and while it seems easy to guide users to not send non-hand images to the system, there are less obvious limitations: What if the image grey scale is inverted, if there are two hands in the image, if the pose of the hand is non-standard, if some fingers are missing, if there is a very large margin around the hand, etc.?

These considerations lead us to propose that an AI system for bone age assessment must have *two* intelligent functions.

(A) An intelligence that *positively* asserts that the system can process the image reliably
(B) An intelligence that derives the bone age value

BoneXpert implements both functions, and function A has been made freely available at www.bonexpert.com/online for readers to assess it on their own images, and verify that for instance images of shoulder and thorax, as well as grey-scale-inverted images, are rejected, and that also some hand images are rejected.

BoneXpert has additional controls to manage the risk associated with deviating image quality. Modern X-rays have typically been subjected to some edge enhancement by the image post-processing software associated with the modality. If the image has been subjected to a stronger degree of edge enhancement than covered by the training data, the rejection type 2—“too sharp”—will arise. There is also a rejection type 3—“too blurred”. These rejections are understandable, so that the radiographer can take actions to avoid them in future, e.g. by adopting milder postprocessing protocol.

It is hoped that the selfvalidation mechanism in BoneXpert can serve as a paradigm for other AI systems in radiology. Current deep learning methods for bone age assessment completely lack such a mechanism.

### Advantages of analysing individual bones – explainable AI

Bone age assessment is particularly well-suited for automation by AI, because of the redundancy of information in the hand radiograph: There are 21 tubular bones, whose maturation are controlled by the same endocrinological processes (the seven carpals have no epiphyses and mature by a different endocrine control—their maturation is less sensitive to sex steroids). As detailed in the supplementary material, BoneXpert implements separate bone age models for each tubular bone, all trained to predict the overall bone age of the hand. Therefore, one can afford to use only a subset of the 21 bones, and still provide an unbiased bone age estimate. Bones can become excluded if they are (partly) outside the radiograph, (partly) occluded by some material, wrongly posed, or malformed. For instance, a prematurely fused fifth middle phalanx (a common normal variant) should be left out of the estimate, and in BoneXpert, such a bone is excluded either because of its odd shape or because the bone age derived from it deviates by more than 2.4 y from the overall average.

This redundancy enables BoneXpert to avoid giving unreliable results by rejecting images with less than 8 accepted bones. Ironically, it is also this redundancy that makes bone age rating disliked by some radiologists: Reliable manual rating requires that each bone is considered in turn so that an average can be formed, and this repetitive work is tedious and time-consuming.

The new deep learning-based bone age methods analyse the hand as a whole, in one giant computational step^6,22^ without any intermediate results. Although these methods also exploit the redundancy of information, they lack the ability to explain the result as an average of individual bone ages—the systems act like a black box. This is a problem, because “the bone age of a hand” is an abstraction, and a bone age method should always be able to trace the overall bone age to the bone ages of individual bones.

There are several advantages of reconstructing the bone borders:

- The GP atlas^1^ (as well as all other bone age methods) uses line-drawings to describe the maturity stages of each bone, so when BoneXpert locates the bone border, it establishes a connection to the reference method.
- The bone borders are used as features in BoneXpert’s bone age model, so the borders constitute an intermediate result, which allow the observer to see, to what extent the machine has understood the image correctly.
- The annotations indicate which bones were used, and what were their predicted bone age values. It is an example of an AI system that explains how it derives the result.

### Bone age below 2 y

The previous version of BoneXpert covered only the bone age interval 2.5–19 y of males, and 2–18 y of females. This gave a risk associated with images with bone age below the lower limit. Most of the time, such images were rejected automatically, but not always, and ultimately it was the user’s responsibility to not send too immature cases through BoneXpert. With version 3, the bone age range of BoneXpert has been extended down to neonates, thereby mitigating this risk completely. The new bone age methods based on deep learning are typically trained on data sets with very few cases below 2 years like the RSNA study. As a result these methods are at risk of being less reliable below 2 years. We have validated that with ample training data down to neonates, BoneXpert’s RMS error below 2 y is smaller than at larger bone ages.

### Comparison with deep learning methods

In summary, we have discussed the following advantage of BoneXpert compared to methods based on deep learning:

1. BoneXpert’s accuracy is MAD 4.1 months, compared to 4.9 months for a prominent deep learning method^19^.
2. BoneXpert has selfvalidation, which is highly desirable and a prerequisite for use as autonomous AI
3. BoneXpert is “explained AI” by means of the individual bone analyses
4. BoneXpert gives reliable bone age also below 2 y

The rejection of 0.4% of the images represents an inefficiency of the BoneXpert method, but many users consider it an advantage, because these rejected images are usually abnormal and they prefer to rate such images manually, and these cases may also require diagnosis of an underlying disease. Likewise, the cases with several rejected bones, as those in Figs. 6, 7, alert radiologists to abnormalities that require their attention.

## Conclusion

Automated bone age rating is now so accurate, that the observed deviation from a single manual rating is dominated by the standard deviation of manual ratings, which in this study ranged from 0.52 to 0.68 y. Therefore, our most reliable assessment of the accuracy of the new version of BoneXpert was made on the RSNA test set of 200 images rated by six raters. The new version obtained an RMS error of 0.45 y against the average. We recommend that future validation studies compare automated methods to the average of three or more raters. This will reduce the error of the "reference", and at the same time allow an estimate of the interrater variability.

We found that the method’s self-validation rejected 0.4% of the images. Among the accepted images there were extremely few outliers: Only six cases deviated by more than 1.5 y from a reference in a search among 8250 images—in comparison we found 72 such deviations of manual ratings.

The method alleviates tedious manual work, while at the same time leaves the difficult examples to expert ratings—this scheme is appealing to radiologist—they have a deep understanding that radiologists should not be completely replaced.

Given these advances, one can no longer consider a single manual rating to be the best clinical practice for bone age evaluation. BoneXpert provides a rapid, accurate and precise measure of bone age, giving the paediatric radiologist time to focus on other aspects of the image, where the radiologist can add value.

## Supplementary Information

Supplementary Information.

## Data Availability

The RSNA data, 14036 images for training and 200 images for testing, are available from RSNA: https://www.rsna.org/education/ai-resources-and-training/ai-image-challenge/rsna-pediatric-bone-age-challenge-2017, accessed 24 Jan. 2022. The Los Angeles data are available from University of Southern California: https://ipilab.usc.edu/computer-aided-bone-age-assessment-of-children-using-a-digital-hand-atlas-2/, accessed 24 Jan. 2022.
