# Supplementary material for: Accuracy and self-validation of automated bone age determination
Source: Sci Rep. 2022 Apr 16;12:6388. doi: 10.1038/s41598-022-10292-y (PMC9013398; doi:10.1038/s41598-022-10292-y)
Supplement: Supplementary file 1 — Supplementary Information. [file 41598_2022_10292_MOESM1_ESM.docx]

## Supplementary material to

Accuracy and self-validation of automated bone age determination

Martin DD, Calder AD, Ranke MB, Binder, G, Thodberg HH

Scientific Reports, revised March 11, 2022

## Determination of bone borders

BoneXpert is based on machine learning, and the image analysis is divided into computational steps, the first being to locate each bone by reconstructing its border. The same model is used for both genders. The reconstruction method is learned from a training set with typically 100 examples with manually annotated bone borders.

We construct statistical models of the shape variation of each bone’s border using principal component analysis ^1^. In addition, we construct shape models of selected groups of bones: metacarpal 2-5, radius and ulna, and the set of carpals. We divide the models into maturity epochs, mainly to accommodate the change of topology of the bone border as the bones mature. For the **tubular** bones (i.e. the ones that form epiphyses), the following four epochs are used – the approximate male bone age ranges are indicated:

- There is no epiphysis, 0-2 y
- There is a separate epiphysis of width ≤50% of the diaphysis, 2-4 y
- There is a separate epiphysis of width **>**50% of the diaphysis, 4-13 y
- The epiphysis is partly or completely fused, 13-19 y

There are up to seven **carpals** (pisiform and triquetrum overlap, so they are treated as one “carpal”). We divide the modelling of the carpals into four epochs:

- Two carpals (capitate and hamate), 0-2 y
- Three carpals (pisiform has appeared), 2-3 y
- Four carpals (lunate has appeared), 3-4 y
- All seven carpals, 4-11 y

In these models, we allow one or more carpals to be missing, so that any number of carpals can result from the reconstruction, and the carpals don’t need to appear in the usual order.

Above a bone age of approximately 11 y (for males), the carpals overlap too much for the method to obtain a sufficient goodness of fit, and the method abandons to determine carpal bone age. Also, if no carpals are found (e.g. for new-born boys), the method reports no carpal bone age.

## Models for prediction of bone age

For each of the 28 bones in the hand, we form a model that predicts a bone age value for that bone.

For the youngest epoch of the tubular bones, and for *all* epochs of the carpals, the bone age models are based solely on the principal components of the shape of the bone border. We train linear models of the first 12 components to predict the *age* in the Paris Study. We transform the ages of the females to the male age scale by a suitably tuned mapping, so that males and females can be pooled in the same modelling. As a second step, the outputs of the linear models are transformed non-linearly to agree with the GP bone age scale of males and females for the images in the GP atlas.

For the three more mature epochs of the tubular bones, the bone age models use a wider set of features as first described in ^2^. In addition to 20 principal components of the shape, a principal component analysis of the intensity image of the bone is performed, and the 20 first scores are used as features. Finally, a Gabor-filtered texture image is formed to capture e.g. features of growth zone fusion, and 20 principal components are extracted. Linear bone age models are trained to predict the over all manual bone age from the scores of shape, intensity and texture, a total of 60 inputs. A non-linear model is applied to the outputs, resulting in a total of 63 parameters.

These models are trained on the six studies with manual GP ratings listed in Table 1 of the paper with a total of 24.000 cases. Separate models are made for males and females, so the average number of training examples per gender and per epoch is about 4000, so when fitting 63 parameters, there is a comfortable ratio of tuned parameters to examples of approx. 1/60. Since the models are linear, it is fast to perform a cross-validation of the models, i.e. train on all cases except 1%, to estimate the performance on the 1% unseen data from the same population. This is repeated 100 times to test on all cases. Such a cross-validation shows that the RMS error is about 1/60’th larger when the model is cross-validated than when the data are part of the training set. This agrees with a general property of linear regression: The relationship between standard error of prediction SEP and standard error of training SET when tuning *p* parameters using *N* examples is

SEP = sqrt((N + p) / (N – p)) * SET ≈ (1 + p/N) * SET

It is appealing that there is this good understanding of the difference between training and test error. This is a result of using a type of machine learning with much fewer parameters than used in modern convolutional neural networks. The formula above has also been generalised to non-linear models ^3^.

The training minimises the RMS error, so it is quite sensitive to cases with large bone age deviations. For the RSNA data, cases which deviate by more than 2.2 years (y), are excluded. This was found to improve the model’s ability to generalise. For the rest of the studies, they had already been studied with the previous version, and in these studies, a small number of cases were found to deviate, and they were rerated to form more reliable bone age value. In the training, we use the corrected values, and table 1 reports the RMS errors for these *curated* data.

A special model is used at the end of puberty for radius and ulna. This was developed prior to this work as described in ^4^. This model was trained on age of normal subjects and then tuned to GP bone age at the end.

In the output of the method, shown in Figure 2 in the main text, the overall bone age is defined from the 21 tubular bones. Carpal bone age is given as a separate number, typically determined only up to bone age 11 y for males, and bone age 9 for females. The best fit to the GP values in the training set was obtained by a weighted average of tubular and carpal bone age with 10% weight on carpals. The entire image analysis is independent of magnification, contrast and brightness of the images, and independent of rotation of the hand in the image plane. The method looks for both left and right hands in the image, and if there are two hands, it analyses the one with the best appearance according to the self-validation.

## Accuracy of automated bone age determination below 2 years

The new version of BoneXpert extends the bone age range down to neonates, whereas the previous version only reached down to males of bone age 2.0-2.5 y and females down to 1.5-2 y. It is therefore particularly relevant to validate the accuracy of the new version at the lowest bones ages and the main text presents a validation in the bone age range 0-2 y in Figure 5. This section provides the details of this validation.

In the male bone age range 0-2 y, we are in the first “epoch” (see above), where the tubular bone age models are trained on the Paris study and the GP atlas. On the female bone age scale, this epoch covers the bone age range 0-1.6 y. So to a good approximation, the bone age models are not trained on data from the RSNA, Tubingen, Erasmus and Los Angeles studies, and we can therefore use these data to perform an independent validation of the accuracy of the bone age determination below 2 y. Figure 5 in the main text shows the Bland-Altman plot for the agreement between BoneXpert and manual bone age in this range. The data in this plot come from the three studies listed in table 1 (the Erasmus study has no images at such low bone age).

*Table 1: The data used for validating automated bone age determination below 2 years*

| Study | Number of images in Figure 5 |
| --- | --- |
| RSNA | 120 |
| The Tubingen studies | 217 |
| The Los Angeles study | 61 |

The RMS error is 0.41 y for boys and 0.33 y for girls below 2 y. The RMS error for the whole bone age range, averaged over genders, and averaged among the studies according to the proportions in Table 1, is 0.61 y on the training set, i.e. 0.62 y cross validated, so the observed RMS errors below 2 y are much smaller. The better accuracy can be understood as a result of maturity indicators changing more rapidly at these low ages – thus the GP atlas has 8 plates from 0-2 y for each gender, while at higher bone ages, there are plates every year or half-year.

The RMS errors below 2 y are lower for females than for males. This can be understood from the sexual dimorphism in the speed of change of maturity indicators. Males at age 2.5 y have on average the same morphology as females at age 2.0, so the speed of change is of males is 0.8 times the speed in females (assuming that males and females have the same morphology, on average, at birth, i.e. disregarding that neonate males are “delayed” by approximately 1 month). Since bone age quantifies morphological changes, we can understand that the RMS error for males is 1/0.8 times that of females.

It is concluded that the accuracy of automated bone age assessment below 2 is considerably better than at higher bone ages, although the validation below 0.5 y is rather scarce.

## References

1. Cootes, T. & Taylor, C. Statistical models of appearance for medical image analysis and computer vision. *Med. Imaging 2001* 236–248 (2001) doi:10.1117/12.431093.

2. Thodberg, H. H., Kreiborg, S., Juul, A. & Pedersen, K. D. The BoneXpert method for automated determination of skeletal maturity. *IEEE Trans.Med.Imaging* **28**, 52–66 (2009).

3. Moody, J. E. The effective number of parameters: An analysis of generalization and reg-ularization in nonlinear learning systems. in *Advances in Neural Information Processing Systems 4, Morgan Kaufmann Publishers, San Mateo, CA* (ed. J. Moody, S. Hanson, R. L. (Eds. .) (Morgan Kaufmann Publishers, Morgan Kaufmann Publishers, San Mateo, CA, 1992).

4. Thodberg, H. H., van Rijn, R. R., Jenni, O. G. & Martin, D. D. Automated determination of bone age from hand X-rays at the end of puberty and its applicability for age estimation. *Int. J. Legal Med.* 1–10 (2016) doi:10.1007/s00414-016-1471-8.
